# Utilising abattoir sero-surveillance for high-impact and zoonotic pig diseases in Lao PDR

**DOI:** 10.1017/S095026882300016X

**Published:** 2023-02-08

**Authors:** Nina Matsumoto, Bounlom Douangngeun, Watthana Theppangna, Syseng Khounsy, Phouvong Phommachanh, Jenny-Ann Toribio, Russell D. Bush, Paul W. Selleck, Laurence J. Gleeson, Jarunee Siengsanan-Lamont, Stuart D. Blacksell

**Affiliations:** 1 Sydney School of Veterinary Science, The University of Sydney, 425 Werombi Road, Camden, NSW, Australia; 2 Mahidol-Oxford Tropical Medicine Research Unit, Faculty of Tropical Medicine, Mahidol University, 420/6 Rajvithi Rd, Bangkok 10400, Thailand; 3 National Animal Health Laboratory, Department of Livestock and Fisheries, Ministry of Agriculture and Forestry, Souphanouvong Avenue, Sikhottabong District, PO. Box 6644, Vientiane, Lao People's Democratic Republic; 4 Centre for Tropical Medicine & Global Health, Nuffield Department of Medicine, University of Oxford, Oxford, United Kingdom; 5 Lao-Oxford-Mahosot Hospital-Wellcome Trust Research Unit (LOMWRU), Mahosot Hospital, Vientiane, Lao People's Democratic Republic

**Keywords:** Abattoir surveillance, Brucellosis, Classical swine fever, Laos*, Pig diseases, Porcine reproductive and respiratory syndrome, Zoonoses

## Abstract

National disease surveillance systems are essential to a healthy pig industry but can be costly and logistically complex. In 2019, Lao People's Democratic Republic (Lao PDR) piloted an abattoir disease surveillance system to assess for the presence of high impact pig diseases (HIPDs) using serological methods. The Lao Department of Livestock and Fisheries (DLF) identified Classical Swine Fever (CSF), Porcine Respiratory and Reproductive Syndrome (PRRS) and *Brucella suis* as HIPDs of interest for sero-surveillance purposes. Porcine serum samples (*n* = 597) were collected from six Lao abattoirs in March to December of 2019. Serological enzyme-linked immunosorbent assay (ELISA) methods were chosen for their high-throughput and relatively low-costs. The true seroprevalence for CSF and PRRS seropositivity were 68.7%, 95% CI (64.8–72.3) and 39.5%, 95% CI (35.7–43.5), respectively. The results demonstrated no evidence of *Brucella spp.* seroconversion. Lao breed pigs were less likely to be CSF seropositive (*P* < 0.05), whilst pigs slaughtered at <1 year of age were less likely to be PRRS seropositive (*P* < 0.01). The testing methods could not differentiate between seropositivity gained from vaccine or natural infection, and investigators were unable to obtain the vaccine status of the slaughtered pigs from the abattoirs. These results demonstrate that adequate sample sizes are possible from abattoir sero-surveillance and lifetime health traceability is necessary to understand HIPDs in Lao PDR.

## Introduction

Lao People's Democratic Republic (Lao PDR) is a landlocked nation in the Greater Mekong Subregion. Lao PDR shares borders with Thailand, Vietnam, Cambodia, China and Myanmar. Structured animal health surveillance programmes are necessary to meet World Animal Health Organisation (WOAH) member nation requirements, thereby satisfying World Trade Organisation (WTO) requirements to trade animal products on the international market. In 2019, the Lao Department of Livestock and Fisheries (DLF) of the Ministry of Agriculture and Forestry piloted an abattoir-based disease surveillance system to detect high impact pig diseases (HIPDs) and ruminant diseases [1].

The Lao pig industry represents a diverse collection of animal types and management styles, from the smallholder to the commercial operator. Lao traditional breeds include small, dark-haired varieties like the *Moo laat* and commercial operations utilise animals of Landrace/Large White type genetics [2]. Smallholder farms routinely use free-ranging and minimal medical or feed inputs, whilst commercial farms in Lao PDR are identical in appearance to any commercial operation in Asia, Europe or the Americas [3, 4, 5]. Despite this variation, the Lao abattoir system is a useful collection point as all animals that are not home-slaughtered will pass through the same facilities [6]. Abattoir-based surveillance provides a cost-efficient adjunct to field-based surveillance activities in monitoring for the presence and spread of HIPDs amongst marketable pig populations [7].

Many HIPDs are endemic in Lao PDR [3] at low levels resulting in occasional outbreaks, such as Foot-and-Mouth disease (FMD) or Classical Swine Fever (CSF). In a recent sero-survey conducted as part of the aforementioned project from March–December 2019, only 7 of 597 porcine serum samples returned a positive result on FMD NSP ELISA, demonstrating an apparent FMD seroprevalence of 1.3% [6]. Other HIPDs are poorly quantified in Lao PDR, such as Porcine Respiratory and Reproductive Syndrome (PRRS) and *Brucella suis* (Brucellosis) [8, 9]. In this study, the diseases CSF, PRRS and Brucellosis were identified by the Lao DLF and partner organisations as diseases of interest for a high-throughput, abattoir-based surveillance system using low-cost ELISA methods.

*Brucella suis* is a zoonotic pathogen of pigs that spreads through contact with raw pig meat, tissues and body fluids to humans [10]. Brucellosis of swine causes clinical reproductive disease such as abortions or orchitis, and occasionally the development of arthritis [10]. Brucellosis in humans causes fevers, pain, swelling of major organs, including the liver, spleen and testicles [11]. Symptoms can last up to months and cause abortions and foetal malformations in pregnant women [11]. CSF received considerable attention over the past 20 years as a disease that is both high-impact and able to be controlled using effective vaccination programmes [2, 3, 12, 13]. Although the Lao DLF have not reported any cases of CSF to the WOAH since 2010, research conducted in 2011 detected CSF antibodies in 11.2% of village pigs in Savannakhet and Luang Prabang, with low vaccine coverage [3]. PRRS virus (PRRSv) outbreaks can be devastating to a commercial piggery, with losses estimated at EUR 75, 724–650, 090 in European commercial operations [14]. PRRSv causes production losses in breeder and grower herds through respiratory disease and reproductive failure, with clinical signs largely dependent upon the virus strain [15]. However, the highly pathogenic PRRSv strain that emerged in China in 2006 and more recently in Southeast Asia has caused mass mortalities [9, 16, 17]. Less pathogenic strains of PRRS may be underreported in Lao production systems using the current surveillance approach. Previous work suggests the presence of both the highly-pathogenic and low-pathogenic strains in the Lao pig population [3, 9].

The current Lao disease surveillance system relies primarily on disease outbreak reporting by village veterinary workers, as described elsewhere [18]. At official abattoirs, a Provincial Agriculture and Forestry Officer (PAFO) will be on-site during slaughter and processing to observe food safety protocols. However, they do not normally collect samples for disease surveillance. This study is a pilot investigation into the use of abattoir-based surveillance to assess the seroprevalence of HIPDs among pigs in Lao PDR. This pilot study investigates the data quality and seroprevalence results from routine sample collection. The results will inform future studies and policymaking decisions. We expect that seroprevalence will likely vary based on province of origin, breed and age of the animals. Depending on abattoir purchasing and lairage procedures, this may even be affected by the abattoir at which they were slaughtered.

## Materials and methods

### Sample sizes and origins

**Fig. 1.**
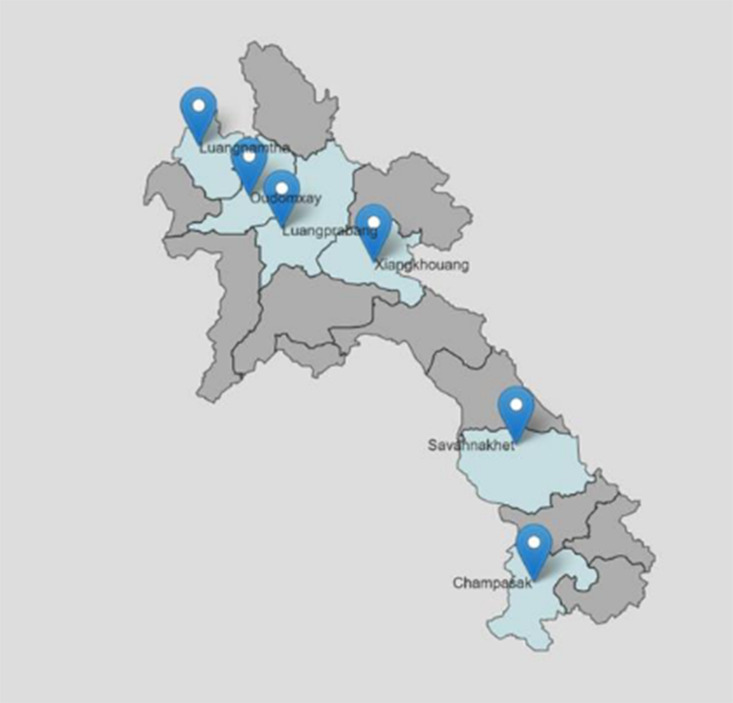
Map of Lao PDR showing provinces included in pilot abattoir sero-surveillance project from March–December 2019 – from Siengsanan-Lamont *et al*. [6].

The sample collection method and sample size justification is detailed in Siengsanan-Lamont *et al*. [1]. The programme was designed for multi-disease surveillance for endemic diseases, across both large ruminants and swine. The USDA simple sample size calculator was used with an estimated disease prevalence of 20%, diagnostic sensitivity of 99% and a confidence level of 95%. Prior work estimated seroprevalence for CSF to be 8.5–13.6% in Lao smallholder owned pigs, whilst there were no prior prevalence estimates for PRRS or Brucellosis. The prevalence was then set at 20%, as the study covered endemic diseases of unknown prevalence. Based on an estimated 30 animals slaughtered per day per abattoir, the adjusted sample size calculated was 11 animals per abattoir when visiting monthly. After consultation and negotiation with the local authorities, this was changed to 10 animals per sample collection round for simplification of shipping and logistics [1]. This method for simple estimation of prevalence does not account for clustering at the abattoir or the point of origin. The serum in this study was collected as part of a pilot abattoir-surveillance programme designed by consultation between the Lao DLF, Lao National Animal Health Laboratory (NAHL) and Mahidol-Oxford Tropical Medicine Research Unit (MORU) with funding provided by the United States – Disease Threat Reduction Agency (US-DTRA). Pilot provinces included Luang Prabang (LPB), Luang Namtha (LNT), Oudomxay (OUD), Savannakhet (SVK), Champasack (CPS) and Xieng Khouang (XK). These provinces were chosen as representative of the northern (LPB, LNT and OUD), central (XK) and southern (SVK, CPS) regions of Lao PDR (Fig. 1).

From March to December 2019 in each selected province, two PAFO and District Animal Health Office (DAFO) staff visited a local abattoir once every month to collect ten jugular blood samples from pigs being slaughtered that day. The abattoirs were chosen by local PAFO and DAFO staff and remained the same throughout the study period. The sample submission questionnaire collected provenance data, age, breed, sex and body condition score (BCS) with a column for brief antemortem health assessment of the animals [19]. The pigs from which whole blood samples were collected at the abattoir were chosen from all available pigs at the time of the field officer's visit to the abattoir. Due to lack of resources and time pressure, these pigs were chosen based on convenience of sampling on the day of collection. The blood was collected into individual sterile syringes and allowed to clot at room temperature. The serum was then decanted into individual marked serum tubes, packed in a styrofoam container with ice and sent back to NAHL by air freight for storage in a dedicated −20-degree Celsius freezer within 5 days of collection.

### Testing methods

All serum samples were tested using the ID Screen^®^ Classical Swine Fever E2 Competition ELISA [20], ID Screen^®^ PRRS Indirect [21] and ID Screen^®^ Brucellosis Serum Indirect Multi-species [22] from IDVet at NAHL. Diagnostic sensitivity and specificity can be found in Table 1. The ELISA assays were performed per the test kit instructions, with optical densities read by a Thermo Fisher Multiskan FC Microplate (ELISA) reader. The optical density of the samples was then used to calculate the signal to positive (S/P) ratio and the signal to negative (S/N) ratio to determine the serological antibody status of each sample (Table 1).

**Table 1.** Details of ELISA test kits utilised in abattoir sero-surveillance pilot study in Laos

Test name	Se	Sp	S/P or S/N calculation (as per instructional leaflet)	Serological cut-off values	Reference
ID Screen^®^ Classical Swine Fever E2 Competition ELISA	1.000	1.000	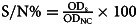	S/P ≤ 50%: Positive	[22]
				S/P 50–60%: Doubtful	
				S/P ≥ 60%: Negative	
ID Screen^®^ PRRS Indirect ELISA	0.998	0.999	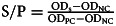	S/P ≤ 0.4: Negative	[23]
				S/P > 0.4: Positive	
ID Screen^®^ Brucellosis Serum Indirect Multi-species ELISA	1.00	1.00	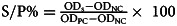	S/P ≤ 110%: Negative	[24]
				S/P 110–120%: Doubtful	
				S/P ≥ 120%: Positive	

Se, sensitivity; Sp, specificity; OD, optical density; _S_, sample; _NC_, negative control; _PC_, positive control; PRRS, Porcine Reproductive and Respiratory Syndrome; S/P, signal to positive ratio; S/N, signal to negative ratio

### Data analysis

The sample information was stored in the Pathogen Asset Control System (PACS) at NAHL, then extracted to Microsoft Excel and RStudio [23] to calculate positive and negative results. Descriptive analysis and logistic regression modelling were performed in RStudio. The true seroprevalence was calculated using the epi.prev() function from the EpiR package, with Wilson's method for the confidence intervals [24].

Due to low numbers of seropositive cases, *Brucella spp.* were excluded from risk factor analysis. CSF and PRRS samples with complete data (*n* = 381) underwent univariate risk factor analysis using the glm() function in RStudio [23]. Available variables are described in Tables 2 and 3. Variables with *P* ≤ 0.2 were included in multivariate logistic regression analysis. Multivariate logistic regression modelling used the glmer() function from the lme4 package in RStudio [25].

**Table 2.** Percentage true seroprevalence of Classical Swine Fever (CSF) and Porcine Respiratory and Reproductive Syndrome (PRRS) in 597 pigs presenting to Lao abattoirs from March–December 2019, grouped by breed and age

	CSF % (95% CI)	PRRS % (95% CI)
Breed		
Exotic	75.2 (70.7–79.2)	43.6 (38.8–48.6)
Mixed	64.5 (46.9–78.9)	61.4 (43.9–76.4)
Native	59.5 (51.4–67)	24.5 (18.2–32)
Age Group (years)		
0–0.5	71.4 (66.8–75.5)	41.9 (37.2–46.7)
0.5–1	54.4 (41.6–66.6)	35.1 (24–48.1)
1–2	58.1 (45.7–69.5)	38.7 (27.6–51.2)
3–4	69.4 (53.1–82)	27.8 (15.8–44)
>4	80 (49–94.3)	40 (16.8–68.8)

CSF, Classical Swine Fever; PRRS, Porcine Reproductive and Respiratory Syndrome

**Table 3.** Results of univariate logistic regression analysis on risk of CSF seropositivity

Variable	Levels	Odds ratio (95% CI)	Std. Error	*P* value
Animal Breed	Exotic^a^	1		
	Mixed	0.59 (0.26–1.44)	1.54	0.22
	Native	0.47 (0.29–0.78)	1.29	<0.01
PRRS Result	Negative^a^	1		
	Positive	1.97 (1.22–3.23)	1.28	0.01
Abattoir	Champasack^a^	1		
	Luang Namtha	0.5 (0.23–1.06)	1.47	0.08
	Luang Prabang	0.92 (0.41–2.09)	1.51	0.85
	Oudomxay	0.92 (0.33–2.84)	1.71	0.88
	Savannakhet	0.5 (0.23–1.08)	1.49	0.08
	Xieng Khouang	0.45 (0.21–0.94)	1.46	0.04
Age Group (years)	>4^a^	1		
	<0.5–1	0.41 (0.22–0.8)	1.39	0.01
	1–2	0.61 (0.31–1.27)	1.43	0.17
	3–4	0.79 (0.39–1.68)	1.45	0.52
Sex	Female^a^	1		
	Male	0.77 (0.49–1.23)	1.27	0.27
BCS^b^	<3^a^	1		
	3	1.24 (0.54–2.69)	1.50	0.59
	≥4	1.26 (0.51–2.93)	1.55	0.60
Region of Origin	Central^a^	1		
	Northern	1.02 (0.55–1.86)	1.36	0.94
	Southern	0.96 (0.53–1.74)	1.36	0.91

CSF, Classical Swine Fever; PRRS, Porcine Reproductive and Respiratory Syndrome; BCS, body condition score

a Denotes referent category

b Animals were classified into BCS 1–2, BCS 3 and BCS 4–5.

Variables with a *P* ≤ 0.05 were retained in a forward stepwise procedure for the final model, with the best fit determined by comparing Akaike information criterion (AIC) values and selecting the model with the lowest AIC value.

The final multivariate model for CSF seropositivity included PRRS result and animal breed as fixed effects, with abattoir of slaughter as a random effect. For the final PRRS model, the region of origin was included as a random effect and fixed effects included abattoir, CSF result and age group. The random effects were chosen due to the respective variable creating a possibility for hierarchical clustering and therefore needing to be accounted for in the model.

## Results

Across all sites, 597 samples were successfully collected, however the field investigators were not able to obtain complete data for pigs at slaughter in some instances. The sample submission questionnaire responses separated by their abattoir of slaughter, are shown in Table 4. The samples collected were mainly from exotic pigs of commercial breed (Large White or Landrace genetics) and will be referred to as exotic from here onwards (399 of 597). Native breed pigs such as *Moo laat* made up a moderate portion of the samples (148 of 597). A few mixed breed and un-labelled pigs also appeared in the sample set (31 and 19 of 597 respectively) (Table 4). Female pigs comprised 260 of the 597 samples, and 176 pigs were male (Table 4). An additional 161 pigs did not have a gender included in their sample submission.

**Table 4.** Descriptive data on 597 pigs sampled at six abattoirs across Laos in 2019

	Champasack (*n* = 100)	Luang Namtha (*n* = 98)	Luang Prabang (*n* = 100)	Oudomxay (*n* = 96)	Savannakhet (*n* = 101)	Xieng Khouang (*n* = 102)
BCS (*n* = 574)						
1	0	0	0	0	2	1
2	4	1	0	0	28	2
3	60	97	54	63	65	24
4	36	0	46	21	5	63
5	0	0	0	2	0	0
Age (years) (*n* = 577)						
0–0.5	55	87	75	82	90	23
0.5–1	1	3	0	2	1	50
1–2	2	8	15	11	0	26
3–4	22	0	0	1	10	3
>4	0	0	10	0	0	0
Breed (*n* = 578)						
Exotic	74	69	75	83	51	47
Mixed	0	20	10	0	0	1
Native	26	9	15	4	40	54
Sex (*n* = 436)						
Female	54	32	55	30	52	37
Male	46	40	15	30	10	35
Province of origin ^(region of origin)^ (*n* = 587)						
Bolikhamxai^C^	0	10	0	0	0	0
Champasack^S^	99	0	0	0	0	0
Luang Namtha^N^	0	46	0	0	0	0
Luang Prabang^N^	0	36	100	34	0	0
Oudomxay^N^	0	0	0	17	0	0
Salavan^S^	1	0	0	0	0	0
Savannakhet^S^	0	0	0	0	101	18
Vientiane Capital^C^	0	0	0	18	0	11
Vientiane Province^C^	0	6	0	17	0	19
Xieng Khouang^C^	0	0	0	0	0	54

^N^, northern region; ^C^, central region; ^S^, southern region; BCS, body condition score

Most pigs were slaughtered at an appropriate BCS for marketing (2–3 out of 5) (Table 4). The animal origins reported by traders to the PAFO represented 11 of the 18 provinces of Lao PDR (*n* = 587), with only ten samples of unlabelled origin. Of the submitted samples, 164 of 597 originated in a province different to their province of slaughter. Despite the question on the sample submission questionnaire, the pigs' vaccination histories were not obtained at slaughter.

### Prevalence of disease seropositivity

#### Classical swine fever seroprevalence

CSF seropositive samples comprised 409 of the submitted 597 samples, with five samples returning doubtful results and a further 183 returning seronegative results. Based on these results, the true prevalence for CSF serological immunity was 68.7% (64.8–72.3) (Table 5). Champasack (*n* = 81 of 100) and Savannakhet (*n* = 58 of 100) abattoirs had the highest (81.0%) and lowest (47.7%) true seroprevalence, respectively. The CSF true seroprevalence by region of origin was 74.9%, 95% CI (68.9–80.0); 65.7%, 95% CI (57.3–73.2) and 66.1%, 95% CI (59.5–72.0) for animals of Northern (*n* = 173 of 231), Central (*n* = 88 of 134) and Southern (*n* = 114 of 218) origin respectively. The seroprevalence by age and breed are shown in Table 2.

**Table 5.** True seroprevalence of CSF, PRRS and Brucellosis based on abattoir surveillance in Laos from March–December 2019

Disease	Seropositive	Seronegative	Doubtful	Apparent prevalence % (95% CI)	True prevalence % (95% CI)
CSF	409	183	5	68.7 (64.8–72.3)	68.7 (64.8–72.3)
PRRS	236	360	NA	39.5 (35.7–43.5)	39.5 (35.7–43.5)
*Brucella suis*	0	597	0	NA^a^	NA^a^

CSF, Classical Swine Fever; PRRS, Porcine Reproductive and Respiratory Syndrome

a Apparent prevalence is less than (1 – Sp). Rogan Gladen estimate of true prevalence is invalid.

#### Porcine respiratory and reproductive syndrome seroprevalence

The PRRS Antibody ELISA returned 236 seropositive samples of the 597 samples. For PRRS virus immunity, true seroprevalence was 39.5%, 95% CI (35.7–43.5) nationally (Table 5). Luang Namtha (*n* = 60 of 98) and Luang Prabang (*n* = 25 of 100) abattoirs had the highest (61.3%) and lowest (25.0%) true seroprevalence, respectively. The PRRS true seroprevalence by region of origin was 39.1%, 95% CI (33.1–45.6); 58.2%, 95% CI (49.7–66.2) and 29.8%, 95% CI (24.1–36.2) for the Northern (*n* = 90 of 230), Central (*n* = 78 of 134) and Southern (*n* = 65 of 218) Regions respectively. The seroprevalence by age and breed are shown in Table 2.

#### Brucellosis seroprevalence

No animals returned a seropositive result to *Brucella spp*. For this reason, Brucellosis was therefore excluded from further statistical analysis in this study (Table 5).

### Risk factor analysis

#### Classical swine fever risk factors

None of the livestock traders surveyed reported the vaccination status (vaccinated or otherwise) of their animals. Region of origin, BCS and gender had no significant influence on CSF seropositivity (Table 3). On multivariate analysis, native breed animals had odds 0.53 times that of exotic breed pigs for being seropositive to CSF. In addition, being PRRS seropositive increased the odds of being CSF seropositive 2.13 times (Table 6).

**Table 6.** Results of multivariable logistic regression analysis on the risk of CSF seropositivity

Variable	Level	Odds Ratio (95% CI)	Std. Error	*P* value
PRRS result	Positive^a^	2.13 (1.22–3.70)	1.33	0.01
Animal Breed	Mixed Breed	0.60 (0.24–1.45)	1.57	0.25
	Native^a^	0.53 (0.31–0.90)	1.31	0.02

CSF, Classical Swine Fever; PRRS, Porcine Reproductive and Respiratory Syndrome

a Indicates result was statistically significant.

#### Porcine respiratory and reproductive syndrome risk factors

Following univariate analysis, all tested variables appeared to have a significant impact upon PRRS seropositivity (Table 7). On multivariate analysis, pigs in the 0.5–1-year age group were protected from PRRS seropositivity with an odds ratio (OR) of 0.3, 95% CI (0.1–0.7) (*P* < 0.001) compared with pigs >4 years of age. Animals slaughtered in the northern provinces, including Luang Namtha (OR 7.2, 95% CI 3.4–15.5), Xieng Khouang (OR 5.6, 95% CI 2.1–14.9) and Oudomxay (OR 4.7, 95% CI 1.8–12.4) were significantly more likely to be seropositive to PRRS (*P* < 0.001) than animals slaughtered in Champasack. CSF seropositive animals had 2.5 times, 95% CI (1.4–4.3), the odds of being PRRS seropositive (*P* < 0.01) (Table 8).

**Table 7.** Results of univariate logistic regression analysis on risk of PRRS seropositivity

Variable	Levels	Odds Ratio (95% CI)	Std. Error	*P* value
Breed	Exotic^a^	1		
	Mixed	1.52 (0.7–3.38)	1.5	0.30
	Native	0.4 (0.24–0.64)	1.3	<0.01
CSF Result	Negative^a^	1		
	Positive	1.97 (1.22–3.23)	1.3	0.01
Abattoir	Champasack^a^	1		
	Luang Namtha	5.58 (2.8–11.5)	1.4	<0.01
	Luang Prabang	0.62 (0.29–1.26)	1.5	0.19
	Oudomxay	4.15 (1.74–10.48)	1.6	<0.01
	Savannakhet	0.69 (0.32–1.45)	1.5	0.34
	Xieng Khouang	2.02 (1.05–3.94)	1.4	0.04
Age group (years)	>4^a^	1		
	<0.5–1	0.64 (0.33–1.2)	1.4	0.18
	1–2	1.21 (0.63–2.31)	1.4	0.56
	3–4	0.55 (0.27–1.06)	1.4	0.08
Sex	Female^a^	1		
	Male	1.63 (1.07–2.47)	1.2	0.02
BCS^b^	<3^a^	1		
	3	3.05 (1.38–7.47)	1.5	0.01
	≥4	1.56 (0.66–4.02)	1.6	0.33
Region of origin	Central^a^	1		
	Northern	0.62 (0.36–1.06)	1.3	0.08
	Southern	0.28 (0.16–0.49)	1.3	<0.01

CSF, Classical Swine Fever; PRRS, Porcine Reproductive and Respiratory Syndrome; BCS, body condition score

a Denotes referent category.

b Animals were classified into BCS 1–2, BCS 3 and BCS 4–5.

**Table 8.** Results of multivariable logistic regression analysis on the risk of PRRS seropositivity

	Odds Ratio (95% CI)	Std. Error	*P* value
Abattoir			
Luang Namtha	7.20 (3.35–15.46)	1.48	<0.01
Luang Prabang	0.62 (0.29–1.32)	1.47	0.21
Oudomxay	4.72 (1.80–12.36)	1.63	<0.01
Savannakhet	0.77 (0.36–1.66)	1.48	0.51
Xieng Khouang	5.64 (2.14–14.89)	1.64	<0.01
CSF result			
Positive	2.48 (1.42–4.32)	1.33	<0.01
Age group (years)			
<0.5–1	0.25 (0.09–0.66)	1.66	<0.01
1–2	0.92 (0.40–2.10)	1.52	0.84
3–4	1.04 (0.49–2.17)	1.46	0.93

## Discussion

This study demonstrates that regularly scheduled abattoir surveillance is achievable given clear guidelines and regular funding. The pilot sites submitted 597 samples of the expected 600 samples. This sample collection schedule is impressive given the presence of ASF from June 2019 onwards in Lao PDR [18]. The ASF outbreak placed strain on local veterinary resources and available pig populations [18]. The question response rate demonstrated informative information can be collected on-site, such as the sex, age and BCS. Unfortunately, liveweight data is difficult to obtain in the Lao abattoir system as weighing facilities are usually reserved for animals after they have been butchered and are being moved to the wet market. The responses also demonstrated that questions relating to the lifetime traceability or histories of the animals, such as specific point of origin or the vaccination status, were not as reliably answered and no plausible explanation for this phenomenon was offered by the Lao field staff. For future studies, investigators might be able to perform additional research to determine the true vaccine status of animals through active surveillance of local pig farms, however there is a need for a government and industry-led programme for traceability in Lao pigs. This can allow abattoir-based surveillance operators to collect this information without having to rely on the traders acting as middlemen between the producer and the abattoir.

This study provides a snapshot of the disease seroprevalence of Lao pigs entering the abattoir system at the same time as the ASF outbreak. Abattoir surveillance is generally biased towards populations of younger, healthier animals [7]. In this study the pigs were generally younger and of exotic breed, without any information collected about antemortem health. This lack of response regarding questions of ante-mortem health is concerning, as the presence of the ASF outbreak during the study suggests that at least some pigs entering the abattoirs must have been either pre-clinical or clinically affected, but is in line with previous work that identified a lack of appropriate ante- and post-mortem inspection in Lao slaughterhouses [26]. The DLF data presented in Xayalath *et al*. [27] suggested that commercial pigs only made up 9% of the total pig population in Lao PDR in 2019, yet they represented 66% of the study population and as such were a biased subset of Lao pigs. Approximately a quarter of the animals travelled across province borders to be slaughtered in this study. This proportion is astonishing, given that half of this study occurred concurrently with the 2019 ASF outbreak. Assuming that the lack of reports of sick animals on antemortem inspection are correct, the movement of trading vehicles and associated staff still presents a risk for national disease transmission.

Lao PDR has numerous opportunities to spread *Brucella suis* from pigs to humans, from the abattoirs and wet markets to the nationally beloved dish of raw pork mince and herbs – *laarp muu* [8, 28]. Our study found no evidence of *Brucella spp.* seroconversion amongst Lao pigs destined for slaughter on antibody indirect ELISA, the test which is recommended by the WOAH Terrestrial Manual for population surveillance [10]. The authors were unable to find any other published data on levels of *Brucella suis* in Lao pig populations, and identify this as a major area for future work. This finding suggests that *Brucella suis* is either not circulating or is circulating at undetectable levels in Lao PDR.

The indirect E2 protein ELISA used in this study could not differentiate between naturally infected and CSF-vaccinated animals [13]. The true seroprevalence of immunity was estimated at 68.7% (64.8–72.3) for CSF. This study included abattoir of slaughter in the modelling as it was identified as a potential source of clustering on study design. When abattoir was included in the logistic regression as a random effect, breed and PRRS status had a significant influence on the CSF status of the animal. These results suggest CSF seropositive animals are more likely to be of commercial origin and have been vaccinated for or exposed to PRRS in their fattening period. These results also show that being of native breeding is correlated with not being exposed to or vaccinated against CSF at the same rate as exotic breed pigs. Native breed pigs are more common in smallholder settings [3], and this trend towards lower serological immunity puts them at risk of outbreaks of CSF in future, threatening smallholder livelihoods and food security [29]. Previous work in Savannakhet and Luang Prabang provinces reported CSF vaccination rates in smallholder settings of 8.5–13.6%, however 59.9% of native pigs returned seropositive results in this survey [3]. This discrepancy suggests that either CSF vaccination rates have increased since Holt *et al*. [3] collected their data or that CSF disease continues to circulate amongst smallholder villages or even a combination of both. Ongoing active surveillance programmes will be of great value in delineating the cause of this increase in serological immunity.

Similar to the CSF ELISA, the PRRS ELISA was unable to differentiate between vaccinated and unvaccinated animals [30], and the local field staff were universally unable to determine the vaccine status of the pigs. The seroprevalence of immunity to PRRS in this survey was estimated at 39.5% (35.7–43.5). Abattoir of slaughter, pig age and CSF status significantly influenced the likelihood of PRRS seropositivity. Where age appeared to have no influence on CSF seropositivity, pigs in the <0.5–1 year old or marketable age category were less likely to be PRRS seropositive. The lack of antemortem data meant that the study was not able to investigate an association between respiratory signs (such as coughing) and PRRS seropositivity. Additionally, reporting of lung lesions at slaughter could help the Lao authorities understand the impact of respiratory diseases such as PRRS on their burgeoning commercial pig industry.

Whilst there appears to be a relationship between CSF seropositivity and PRRS seropositivity, the differences in significant variables in the multivariate analysis are likely due to the differing proportions of animals returning positive CSF and PRRS results. In the univariate analysis both diseases showed significant results in the age group and breed categories. Interestingly, whilst likelihood of CSF seropositivity decreased with age, the opposite trend held with PRRS. This is possibly because of the older sows either being sub-productive (i.e. PRRS affected) or more likely to be vaccinated than grower populations, however further research is necessary for the Lao pork industry to manage this disease risk more effectively.

Research into the pig purchasing preferences of abattoirs could shed light on the association between serological results and abattoirs. Abattoirs could preferentially purchase from producers who perform prior vaccination of their pigs, or perhaps abattoirs are choosing to buy from producers or middlemen that source consistently higher carcass yield animals which are of exotic genetics and fully vaccinated against endemic and production limiting diseases. Understanding the liveweight or dressed weight of these animals at slaughter may assist in future analyses of these animals' serological results.

Whilst CSF vaccine is imported and used routinely in Lao PDR, the vaccine for PRRS is not currently routinely used in Lao pork operations (S. Khounsy, written comm. 2021). The exception being some multinational large-scale operations that privately import the PRRS vaccine for use in country (S. Khounsy, written comm. 2021). Further investigation into the presence of low-pathogenic or highly pathogenic PRRS in the Lao commercial pig industry is warranted, as PRRS vaccine may need to be imported routinely to promote future growth in this transitional economy.

The presence of true infections or exposures to CSF and PRRS in the pigs is of concern due to health and welfare implications and the financial losses incurred by the diseases, as well as the food safety implications of disease-affected animals entering the food chain. Previous work investigating the knowledge, attitudes and practices of slaughterhouse workers in Lao PDR raised concerns about the conditions within abattoirs not meeting international food safety or animal welfare standards [26]. This justifies ongoing work strengthening abattoir surveillance and personnel capacity in Lao PDR.

A robust and representative array of national animal disease surveillance programmes are required to meet the requirements of WOAH member nation. Therefore the motivation exists to pursue the implementation of programme such as this. Based on the results achieved, the sample sizes are adequate to contribute to estimates of the seroprevalence of a disease. However, further research and governmental organisation are required to provide more confidence about the spatial distribution of disease. Furthermore, lifetime traceability documents would assist in accurately tracing pork products, allowing the separation of vaccinated from naturally infected animals in future serosurveys. This study demonstrated the feasibility and initial outputs of a pilot abattoir surveillance system in Lao PDR, and based on the results of this study the work was extended to all provinces at the end of 2019.

## Data Availability

Readers may contact the corresponding authors if they wish to access the resources that support our findings.
